# *Drosophila* as a Rapid Screening Model to Evaluate the Hypoglycemic Effects of Dipeptidyl Peptidase 4 (DPP4) Inhibitors: High Evolutionary Conservation of DPP4

**DOI:** 10.3390/biomedicines11113032

**Published:** 2023-11-12

**Authors:** Francisco Alejandro Lagunas-Rangel, Sifang Liao, Michael J. Williams, Vladimir Trukhan, Robert Fredriksson, Helgi B. Schiöth

**Affiliations:** 1 Department of Surgical Sciences, Functional Pharmacology and Neuroscience, Uppsala University, 751 24 Uppsala, Sweden; francisco.lagunas@neuro.uu.se (F.A.L.-R.); sifang.liao@neuro.uu.se (S.L.); michael.williams@uu.se (M.J.W.); 2 Advanced Molecular Technology LLC, 354 340 Moscow, Russia; vladimir.trukhan@gmail.com; 3 Department of Pharmaceutical Biosciences, Uppsala University, 751 24 Uppsala, Sweden; robert.fredriksson@uu.se

**Keywords:** diabetes, alternative animal model, new drugs, glucose levels

## Abstract

Dipeptidyl peptidase 4 (DPP4) inhibitors, commonly known as gliptins, have been an integral part of the treatment of type 2 diabetes mellitus (T2DM) for several years. Despite their remarkable efficacy in lowering glucose levels and their compatibility with other hypoglycemic drugs, recent studies have revealed adverse effects, prompting the search for improved drugs within this category, which has required the use of animal models to verify the hypoglycemic effects of these compounds. Currently, in many countries the use of mammals is being significantly restricted, as well as cost prohibitive, and alternative in vivo approaches have been encouraged. In this sense, *Drosophila* has emerged as a promising alternative for several compelling reasons: it is cost-effective, offers high experimental throughput, is genetically manipulable, and allows the assessment of multigenerational effects, among other advantages. In this study, we present evidence that diprotin A, a DPP4 inhibitor, effectively reduces glucose levels in *Drosophila* hemolymph. This discovery underscores the potential of *Drosophila* as an initial screening tool for novel compounds directed against DPP4 enzymatic activity.

## 1. Introduction

Human CD26/dipeptidyl peptidase 4 (DPP4) is a membrane-bound glycoprotein consisting of 766 amino acids and comprising four distinct regions: an N-terminal short cytoplasmic domain (residues 1–6), a transmembrane domain (residues 7–28), a flexible region (residues 29–39), and a C-terminal extracellular domain (residues 40–766) containing peptidase activity [1]. This protein is found in many types of cells in the body, and a circulating soluble form can also be found [2]. DPP4 functions as an exopeptidase with a particular penchant for substrates containing proline or alanine in the penultimate position at the N-terminal end. Consequently, this enzyme exhibits selectivity in the degradation of a diverse array of substrates, including incretin hormones, growth factors, and cytokines [3].

DPP4 inhibitors, commonly referred to as gliptins, have been a pivotal component in the treatment of type 2 diabetes mellitus (T2DM) since their introduction in 2006 [4]. Unlike many other antidiabetic drugs, these drugs do not possess inherent hypoglycemic properties. Instead, their efficacy as antidiabetic agents is closely related to their ability to protect specific DPP4 substrates from degradation. These substrates include critical molecules, such as the incretin hormones (e.g., glucagon-like peptide 1 [GLP-1] and glucose-dependent insulinotropic polypeptide [GIP]), as well as neuropeptides, like neuropeptide Y (NPY) and peptide YY (PYY), and the pituitary adenylate cyclase-activating polypeptide (PACAP), among others [3,5]. By preserving the stability of these substrates, DPP4 inhibitors facilitate various beneficial effects, including the stimulation of insulin synthesis and the inhibition of glucagon secretion. They also exert appetite-suppressing effects, contribute to weight reduction, slow down gastric emptying, and to some extent, help restore the functionality of pancreatic β-cells [3,5,6,7].

Currently, the US Food and Drug Administration (FDA) has approved five DPP4 inhibitors for the management of T2DM, namely sitagliptin, vildagliptin, saxagliptin, alogliptin, and linagliptin [8,9]. These DPP4 inhibitors stand out for their user-friendly characteristics, as they are well-tolerated oral antihyperglycemic medications with high bioavailability. Importantly, they do not necessitate dose adjustments and can be taken at any time of the day, regardless of meals [10]. Furthermore, because their mechanism of action involves both insulinotropic and glucagonostatic effects, they can easily be combined with other antidiabetic agents for increased efficacy [5]. In recent years, the physiological profiles and clinical properties of each DPP4 inhibitor, as well as the side effects that these drugs may cause, have been extensively and intensively studied [11,12,13,14]. Thus, placebo-controlled clinical trials have shown that some of the DPP4 inhibitors cause a small increase in hospitalizations due to heart failure [13,15], the incidence of acute pancreatitis [16,17,18], and arthralgia [19,20,21]. 

The evaluation of new drugs requires the use of experimental animals to examine various pharmacokinetic and pharmacodynamic attributes before moving to clinical trials. Nevertheless, there is increasing interest in minimizing reliance on mammals whenever possible, advocating for alternative animal models [22]. 

*Drosophila*, commonly known as the fruit fly, is an organism that has become a robust model for high-throughput screening of new drugs. Its fast generation times and cost-effectiveness allow the use of a considerable number of specimens, which increases the robustness and statistical significance of screening results [23]. Furthermore, *Drosophila* genetics have been extensively studied and exhibit metabolic pathways similar to humans, enabling the identification of potential drug targets via mutant analysis [24]. Due to its short generational time, *Drosophila* also facilitates the assessment of multigenerational drug effects [25]. Previous studies have suggested that many *Drosophila* proteins show substantial evolutionary conservation compared with human proteins, reflecting similarities in protein structure and function, as well as the presence of conserved domains or specific sites that underscore their functional roles throughout evolution [26,27]. In light of all these considerations, our study aimed to determine whether *Drosophila*, an organism with previously documented DPP4 activity [28,29], could serve as a suitable model for assessing the hypoglycemic effects of a DPP4 inhibitor, such as diprotin A. In order to better understand the conservation of DPP4, we performed substantial studies of the evolution and sequence similarities of DPP4 between different species. These findings propose the use of the fruit fly as a preliminary screening tool for novel DPP4 inhibitory compounds, potentially reducing the need for extensive experiments with mice, rats, or other mammals.

## 2. Materials and Methods

### 2.1. Sequences

To find orthologs of the human DPP4 protein in other species, we used the NCBI BLAST tool with predetermined search parameters. The complete sequences of the DPP4 orthologs were obtained from the UniProt Knowledgebase (UniProtKB) [30] and these include those of the following species: *Homo sapiens* (P27487), *Mus musculus* (P28843), *Rattus norvegicus* (P14740), *Sus scrofa* (P22411), *Bos taurus* (P81425), *Felis catus* (Q9N2I7), *Canis lupus* (A0A8C0NCU9), *Aspergillus oryzae* (Q2UH35), *Aspergillus clavatus* (A1CHP1), *Neosartorya fischeri* (A1CX29), *Trichophyton rubrum* (Q5J6J3), *Trichophyton verrocosum* (D4CZ59), *Trichophyton equinum* (A7UKV8), *Trichophyton tonsurans* (B6V868), *Arthroderma otae* (A0S5V9), *Monodelphis domestica* (K7DYU6), *Danio rerio* (B5DDZ4), *Capra hircus* (A0A452FGS0), *Cavia porcellus* (A0A286XN52), *Heterocephalus glaber* (A0A0P6J3T0), *Nomascus leucogenys* (G1QQ71), *Ovis aries* (W5P906), *Mustela putorius* (M3XN99), *Pan troglodytes* (H2R124), *Macaca mulatta* (F6VRB0), *Gorilla gorilla* (G3SI68), *Cercocebus atys* (A0A2K5LB00), *Ailuropoda melonoleuca* (G1LG48), *Propithecus coquereli* (A0A2K6GYP1), *Loxodonta africana* (G3TVN4), *Physeter macrocephalus* (A0A2Y9EMH4), *Delphinapterus leucas* (A0A2Y9N2E9), *Balaenoptera musculus* (A0A8C0I050), *Equus caballus* (A0A3Q2HL58), *Rhinolophus ferrumequinum* (A0A2Z5CWD5), *Carollia perspicillata* (A0A2Z5CWD9), *Saccopteryx bilineata* (A0A2Z5CWB7), *Epomops buettikoferi* (A0A2Z5CWD4), *Macronycteris gigas* (A0A2Z5CWD8), *Rousettus aegyptiacus* (A0A2Z5CWB8), *Artibeus planirostris* (A0A2Z5CWB9), *Crocodylus porosus* (A0A7M4FPI9), *Python bivittatus* (A0A9F2Q2Y6), *Ophiophagus hannan* (V8P9G9), *Gallus gallus* (A0A1D5PJA5), *Drosophila melanogaster* (Q29R16), *Pseudonaja textilis* (A0A670YVH3), and *Lepidothrix coronata* (A0A6J0IYQ4).

### 2.2. Domain Analysis, Alignments, Phylogenetic Analysis, and Protein Modeling

The structural domains of the DDP4 proteins from all organisms studied were predicted and analyzed using InterPro [31]. All the sequences obtained were aligned with the MEGA11 software version 11.0.10 [32] using the MUSCLE algorithm and then manually edited after visual inspection. Conservation calculations were performed with CLC Genomics Workbench version 23.0.5 (QIAGEN Digital Insights). The aligned sequences were used to construct the phylogenetic trees using maximum likelihood and neighbor joining methods. A bootstrap consensus tree was performed that assesses the robustness of the nodes with 100 replicas and 10 random addition heuristic searches for each node. The 3D models of the human and *Drosophila* DPP4 proteins were predicted with AlphaFold version 2.1.1 [33], and illustrations were made using UCSF Chimera software version 1.17.3 [34]. The quality of the generated 3D structures was rigorously assessed using the validation program PROCHECK [35]. This evaluation involved a meticulous examination of the Psi/Phi angles within a Ramachandran plot.

### 2.3. Fly Strains and Maintenance

The *CSORC* wild-type lab strain was created by crossing wild-type strain *Canton S* and *Oregon-R-C* flies that were obtained from the Bloomington Stock Center. All fly strains were maintained with Jazz-Mix *Drosophila* food (Thermo-Fisher Scientific, Waltham, MA, USA, AS153) supplemented with 1.5% yeast extract (Apex Bioresearch Products, San Diego, CA, USA, 20-254), 0.3% propionic acid (Sigma-Aldrich, St. Louis, MI, USA, 402907), and 0.05% tegosept (Apex Bioresearch Products, 20-258). The flies were reared at 25 °C and 60% humidity in a 12:12 h light/dark cycle.

### 2.4. Treatment of Flies with Drugs

Diprotin A (Sigma-Aldrich, D3822) was prepared by dissolving it in DMSO to achieve a concentration of 10 mM. Subsequently, these stock solutions were further diluted into the fly food immediately before application. The standard fly food was mixed with Diprotin A to create food formulations containing concentrations of 5 µM, 15 µM, and 45 µM of the compound. For experimentation, three-day-old adult male flies were introduced into vials containing either the regular fly food or the food enriched with the respective drug concentrations. These vials were then incubated for a period of 3 days under the designated conditions, as described previously. In this experiment, a cohort of 30 flies was used.

### 2.5. Capillary Feeding (CAFE) Assay

This method was modified from Ja et al., 2007 [36]. Individual flies were carefully placed in 1.5 mL Eppendorf microcentrifuge tubes, each equipped with an inserted capillary tube (5 μL, Sigma). To assess feed intake, the capillary tube was filled with a liquid diet consisting of 5% sucrose, 2% yeast extract, and 0.1% propionic acid. To assess water consumption in relation to food, the flies were provided with a capillary tube filled with Milli-Q water in addition to the capillary tube containing food. As controls to account for evaporation, Eppendorf tubes with food and water capillaries but no flies were included. The final measurement of food intake or water consumption was calculated by subtracting the decrease in food or water levels from the mean decrease in the control capillaries. Food consumption was monitored daily and calculated cumulatively for four consecutive days, using 8 to 10 three-day-old adult male flies for each of the three biological replicates. The entire experimental setup was maintained at a constant temperature of 25 °C in a 12:12 h light/dark cycle.

### 2.6. Fly Starvation Assay

To assess resistance to starvation, we employed the *Drosophila* Activity Monitoring System (DAMS) provided by TriKinetics (Waltham, MA, USA). Following a 3-day period of feeding with either standard food or food containing the drug of interest, three-day-old individual flies were transferred to 5 mm diameter tubes for monitoring with the DAMS system. These tubes were filled with 1% agarose (~2–3 cm high). The agarose served to provide water and moisture to the flies but did not act as an energy source during the starvation test. A black cap was then placed on the end containing agarose and the flies were introduced through the open end. After placing the flies in the tubes, the opening was sealed with a cotton plug. All tubes were placed at 25 °C on monitors that determine the live/dead status of flies by beam crossing numbers. Thirty flies were examined for each drug and concentration, with data collected from two independent replicates. Starvation resistance was then determined by calculating the mean survival time under conditions of starvation.

### 2.7. Measurement of Fly Hemolymph Glucose

The concentrations of fly hemolymph glucose were evaluated using Liquick Cor-Glucose diagnostic kit (Cormay, Lublin, Poland, 2-202). Three-day-old flies fed with standard food or food containing the drug of interest were used. They were reared at 25 °C and 60% humidity on a 12:12 h light/dark cycle. At least 6 biological replicas were prepared from both the control groups and the experimental group. For each replica, 10 mg of male flies were decapitated and homogenized in 100 µL of PBS buffer (Gibco, New York, NY, USA, 10010023). Then, samples were centrifuged at 3000 rpm for 6 min, maintaining a temperature of 4 °C. The supernatant was collected and 10 µL was mixed with 650 µL of glucose reaction mix (Cormay, 2-202) in a 96-well plate. The absorbance was measured with a Multiscan GO microplate spectrophotometer (Thermo Scientific) at 500 nm. To produce the standard curve, a serial dilution was made from a 10 µg/µL glucose standard solution. The concentration was calculated according to the standard curve produced.

### 2.8. Measurement of Fly Protein

To evaluate changes in the protein content of flies after drug treatment, at least 6 biological replicas were prepared from both the control groups and the experimental group. For each replica, 10 mg of male flies (3 days old and fed with diprotin A or vehicle [Control]) were decapitated and homogenized in 100 µL of PBST (pH = 7.2, 0.05% Tween 20) with a pellet pestle (Kimble, Dover, OH, USA, 749521-1500) on ice. From this homogenate, 25 µL was taken for measurements using the Pierce BCA protein assay kit (Thermo Scientific, 23225) according to the supplier’s directions. To produce the standard curve, a serial dilution was made from a standard solution of 2 mg/mL bovine serum albumin (BSA). The concentration was calculated according to the standard curve produced.

### 2.9. Measurement of Fly Triglycerides (TAGs)

Five flies were collected for each biological sample and 6 to 8 replicates were prepared for all control and experimental groups. The flies were homogenized in 100 µL of cold PBST (pH = 7.2, 0.05% Tween 20) with a pellet pestle (Kimble, 749521-1500). The homogenate was heated for 10 min at 70 °C and then 20 µL of it was added to two tubes. A total of 20 µL of PBST was added to the first tube to measure free glycerol, while 20 µL of triglyceride reagent (Sigma Aldrich, T2449) was added to the second tube to measure the total glycerol content. All samples were incubated at 37 °C for 60 min and then centrifuged for 3 min at 14,000 rpm. After incubation, 30 µL of each individual sample was transferred to a 96-well plate and mixed with 100 µL of free glycerol reagent (Sigma-Aldrich, F6428). The plate was incubated for 5 min at 37 °C. The absorbance of each sample was measured with a Multiscan GO microplate spectrophotometer (Thermo Scientific) at 540 nm and calculated according to the standard curve. The standard curve was obtained from a serial dilution of glycerol standards (Sigma-Aldrich, G5516) and produced together with the samples. The concentration was calculated according to the standard curve produced and the concentration of TAGs was determined by subtracting the free glycerol concentration from the total glycerol concentration.

### 2.10. Statistical Analysis

The data obtained in each experiment were analyzed with the Shapiro–Wilk test to determine whether they followed a normal or non-normal distribution. For data displaying a normal distribution, statistical analysis was carried out using either Student’s *t*-test or one-way ANOVA, supplemented with a Dunnett post hoc test as necessary. Survival curves were assessed employing the Mantel–Cox log-rank test. GraphPad Prism version 8.0.2 (GraphPad Software, La Jolla, CA, USA) was used to perform the analysis. Data with a *p* value ≤ 0.05 were considered significant.

## 3. Results

### 3.1. DPP4 Is an Evolutionarily Conserved Protein

**Figure 1:**
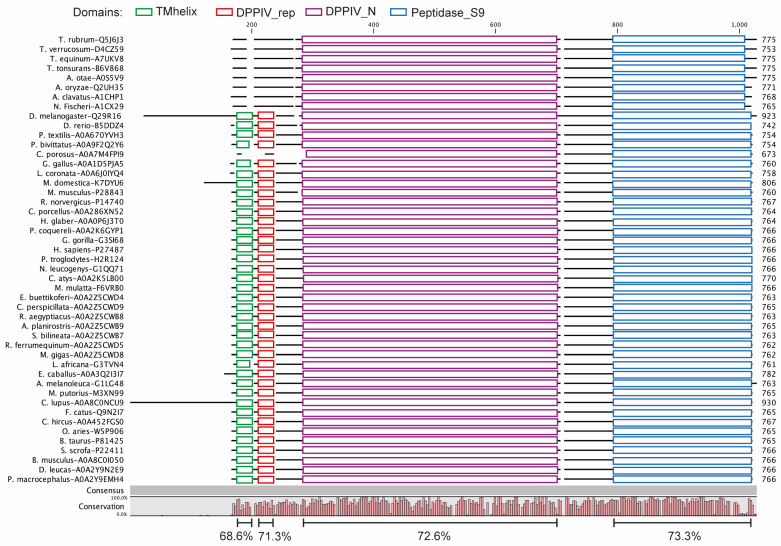
**DPP4 domains in the different species.** Alignment of the amino acid sequences of DPP4 in the different species and their categorization in the different domains. TMhelix represents a conserved transmembrane helix as well as a flanking sequence, DPPIV_rep represents a sequence that can be repeated in the low complexity region between the helical N-terminal and the N-terminal domain of DPPIV, DPPIV_N is an alignment of the region with the N-terminal side of the active site of DPPIV, and finally, Peptidase_S9 covers the serine of the active site of serine peptidases belonging to the MEROPS S9 family of peptidases (prolyl oligopeptidase family, clan SC). DPP IV is an example of a protein possessing this domain. The identity percentages for each domain and global are shown, as well as the conservation plot.

**Figure 2:**
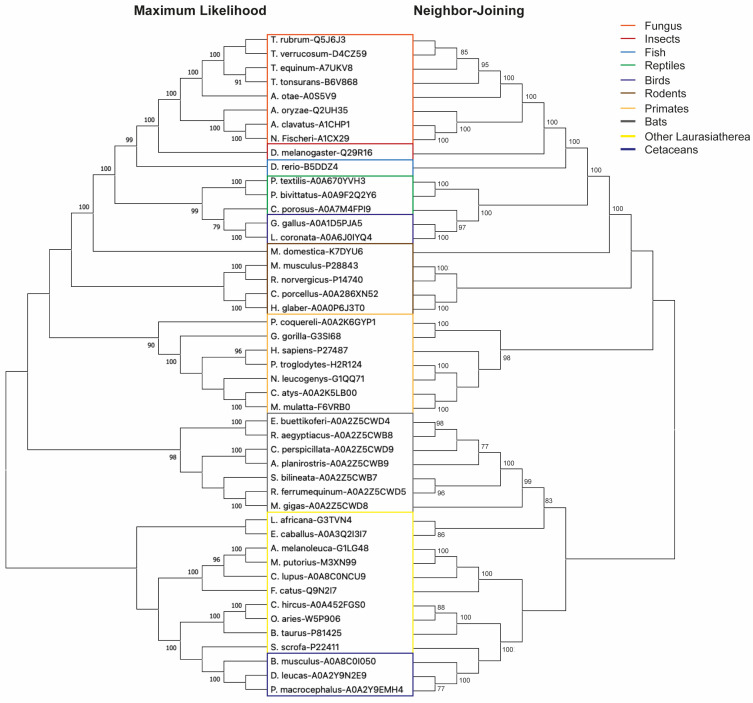
**Phylogenetic analysis of DPP4 orthologs.** The full-length amino acid sequence of DPP-4 orthologs in different species was obtained from UniProtKB [30] and the consensus tree was generated using maximum likelihood and neighbor joining analysis. The numbers before the nodes indicate the percentage of bootstrap replicas supporting that node.

**Figure 3:**
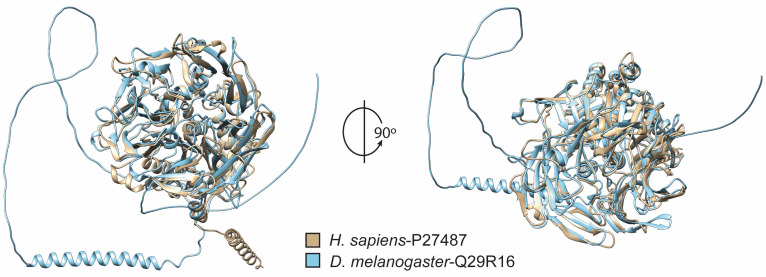
**Overlapping of the extracellular structures of DPP4 modeled for human and *Drosophila* proteins.** The superimposed figures show only slight differences between the human DPP4 protein and the *Drosophila* protein.

In order to explore possible alternative models for evaluating new DPP4 inhibitors, we performed an analysis of orthologous sequences of this protein in several species. Our investigation yielded a collection of DPP4 orthologs spanning 47 different species (Supplementary Figure S1). Notably, the average sequence similarity among this diverse set of orthologs stood at 70.5% (Figure 1). We computed conservation scores for the domains TMhelix, DPPIV_rep, DPPIV_N, and Peptidase_S9. Our analysis revealed identity percentages of 68.6%, 71.3%, 72.6%, and 73.3%, respectively, highlighting the notable degree of conservation within these particular domains. These findings indicate that the conservation of these proteins progressively intensifies towards the C-terminus, concentrating mainly on the catalytic domains of the enzyme. It should be noted that transmembrane domains (TMhelix) were not identified in the sequences of all fungi (Figure 1). The same occurred in the case of the reptile *Crocodylus porosus*, but their absence may be attributed to the fact that their sequence is still under construction and verification. Subsequently, a phylogenetic analysis using maximum likelihood and neighbor-joining methods showed that DPP4 broadly adheres to evolutionary relationships among the included species (Figure 2). From here, we highlight its presence in *Drosophila melanogaster*, a model widely used in aspects of sugar regulation [37]. We predicted the structures of the human and *Drosophila* DPP4 proteins, and by superimposing them, only small differences were observed (Figure 3). The only thing that stands out is the long cytoplasmic domain of the *Drosophila* protein (Figure 1). Both proteins share a common structural organization in their extracellular regions, characterized by a unique eight-bladed β-propeller and α/β-hydrolase.

### 3.2. Diprotin A Does Not Alter Food Intake and Starvation Resistance in Fruit Flies

**Figure 4:**
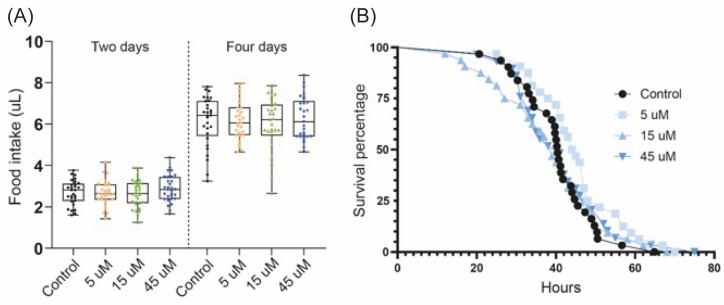
**Diprotin A has no effect on *Drosophila* food intake and starvation resistance.** (**A**) Exposure of flies to 5 µM, 15 µM, and 45 µM diprotin A had no effect on cumulative food intake for two and four days. Thirty flies were used for each treatment and three repetitions were carried out. The data were analyzed by one-way ANOVA with a Dunnett posthoc, thus not finding significant differences between the groups. (**B**) Flies fed 5 µM, 15 µM, and 45 µM diprotin A for three days did not show significant differences in resistance to starvation compared with control flies. Survival curves were evaluated using the Mantel–Cox log-rank test.

We decided to test diprotin A, a drug that has shown hypoglycemic effects in mice [38], in *Drosophila*. First, we fed the flies with three different concentrations of the drug (5 µM, 15 µM and 45 µM) and determined through a CAFE assay if it influenced the cumulative food intake for 2 and 4 days. No significant differences were found with any drug concentration or at any time (Figure 4A).

Subsequently, it was also evaluated whether diprotin A affected the general metabolic status of the flies, for which a starvation resistance assay was performed using the same concentrations. No significant differences were found in the survival of the flies when fed with any dose of the drug compared with the control group (Figure 4B). 

These results collectively suggest that diprotin A does not have an impact on the quantity of food consumed by the flies. Furthermore, it appears to leave their overall metabolic state unaltered, thereby exerting no discernible effect on their survival, neither decreasing nor increasing it.

### 3.3. Diprotin A Reduces Hemolymph Glucose Levels but Not Protein and TAG Content in Fruit Flies

**Figure 5:**
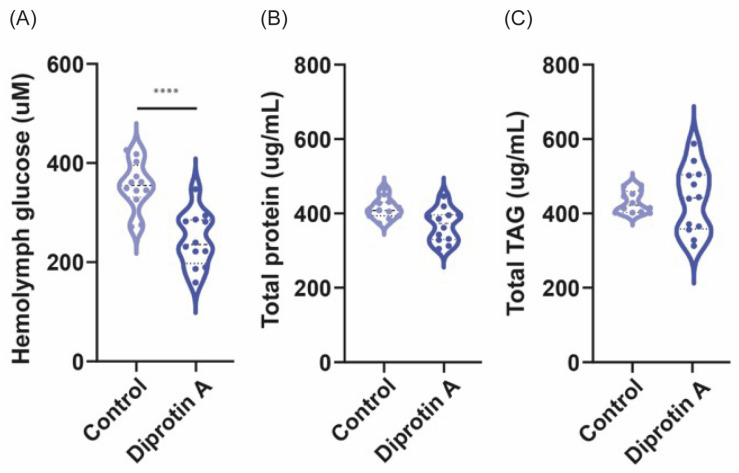
**Diprotin A treatments cause a decrease in glucose levels in the *Drosophila* hemolymph, but do not affect the total content of proteins and TAGs.** (**A**) The flies fed diprotin A significantly reduced their hemolymph glucose levels with respect to the control group. No changes were found in the (**B**) total protein and (**C**) TAG content in the flies. The data were analyzed using Student’s *t* test. Statistical significance is indicated as **** *p* ≤ 0.0001.

Since DPP4 inhibitors improve the body’s own ability to control blood glucose by increasing the active levels of incretin hormones in the body, we decided to evaluate whether diprotin A could reduce the glucose levels in the hemolymph of flies. For this, the flies were fed for three days with 15 µM of the drug and later the glucose content in the hemolymph was determined (see Section 2). A significant decrease in hemolymph glucose levels could be observed with diprotin A compared with control flies (*p* ≤ 0.0001) (Figure 5A). 

We also evaluated whether the drug had an effect on the protein and TAG content of the flies. No significant differences were found in protein (*p* = 0.9) (Figure 5B) or TAG content (*p* = 0.84) (Figure 5C) in flies fed diprotin A compared with the control group. In summary, our findings demonstrate that diprotin A possesses the ability to lower glucose levels in flies without affecting the protein and TAG content. This collective evidence underscores the suitability of *Drosophila* as a valuable screening model to investigate the hypoglycemic effects of this drug class.

## 4. Discussion

Before this study, DPP4 activity had been previously documented in *Drosophila* [28,29] and here, we demonstrate that this protein occupies an evolutionary position intermediate to the DPP4s found in vertebrates and fungi. Other insect DPP4 appear to be in the same evolutionary locality [39]. It is noteworthy that the DPP4 protein shows a substantial level of sequence identity, approximately 70%, between the different species, particularly the domains associated with exopeptidase activity (Figure 1 and Figure S1). 

In fungal species, it was not possible to identify the transmembrane domain. This observation may suggest that the soluble form of this protein was the initial iteration, subsequently undergoing adaptation by anchoring to the cell membrane. This transformation may have occurred when the protein assumed additional non-enzymatic functions, such as participation in cell signaling [40]. In the case of *Crocodylus porosus*, the absence of an identifiable transmembrane domain may primarily be attributed to the ongoing construction and verification process of its sequences. The functional implications of the absence of the transmembrane domain in the fungal DPP4 system are currently unknown. However, this finding suggests the possibility that the pharmacological response to gliptins may vary significantly between fungi and humans, making fungi a potentially more suitable model to study the soluble form of DPP4.

As a general principle, the greater the number of species included in a conservation analysis, the greater its robustness [41]. In our study, we adopted this perspective by including data from 47 different species, making it more comprehensive than a previous study on DPP10, which focused on a smaller set of species [39]. The resulting phylogenetic analysis revealed a gradual transformation consistent with evolutionary patterns, ranging from fungi to mammals (Figure 2). A comparative analysis of the extracellular domains of human and *Drosophila* DPP4 revealed minimal structural disparities (Figure 3). However, it is noteworthy that the fly protein sequence has a more elongated cytoplasmic domain compared with human DPP4 (Figure 1). Although the specific function of this elongated cytoplasmic domain in *Drosophila* remains unknown, it raises the intriguing possibility that it may be implicated in cell signaling or other yet-to-be-discovered functions [28,40]. 

In humans, DPP4 plays a pivotal role in degrading several key substrates, including circulating gut-derived incretins (e.g., GLP-1, GIP, and gastrin-releasing peptide [GRP]), as well as neuropeptides (e.g., PYY, NPY, and Substance P). Additionally, it targets cytokines (e.g., interleukin 3 [IL-3], granulocyte-macrophage colony-stimulating factor [GM-CSF], granulocyte colony stimulating factor [G-CSF], erythropoietin, and fibroblast growth factor 2 [FGF2]) and chemokines (e.g., C-C motif chemokine ligand 2 [CCL2], C-C motif chemokine ligand 3 [CCL3], C-C motif chemokine ligand 11 [CCL11], C-C motif chemokine ligand 22 [CCL22], C-X-C motif chemokine ligand 10 [CXCL10], C-X-C motif chemokine ligand 11 [CXCL11], and C-X-C motif chemokine ligand 12 [CXCL12]) [42]. However, it is worth noting that there might be variations in the repertoire of substrates between mammals and *Drosophila*. Some substrates that are specific to mammal DPP4 may not exist in the *Drosophila* system, and vice versa.

Animal experiments play a crucial role in evaluating various aspects of a new drug, such as dose response, drug toxicity, pharmacokinetics, and pharmacodynamics, before moving on to clinical trials. However, it is important to note that mammalian utilization currently faces significant constraints, arising from a number of factors, including ethical and social concerns [43]. Consequently, there has been a remarkable impetus for the development of alternative approaches to animal experimentation, driven by the application of the 3Rs principle: replacement, reduction, and refinement [44]. Our study aimed to assess whether flies serve as a suitable model for evaluating the hypoglycemic effects of DPP4 inhibitors. Thus, our study demonstrates that a DPP4 inhibitor, such as diprotin A, has the capability to reduce glucose levels in the *Drosophila* hemolymph (Figure 5A), without inducing a significant decline in the total protein or TAG content (Figure 5B,C). This suggests that DPP4 inhibitors exhibit consistent hypoglycemic effects in the fly model, thereby establishing it as a suitable platform for evaluating these drugs. Furthermore, it is noteworthy that diprotin A did not cause a reduction in the amount of food ingested by the flies (Figure 4A), something that was observed in mice that received the DPP4 inhibitors vildagliptin [45] or anagliptin [46]. Likewise, the survival of the flies was not affected when feeding the drug for three days (Figure 4B), which would indicate that whole organisms can tolerate it adequately. Taken together our findings will support the notion that *Drosophila* may serve as an initial screening platform for novel DPP4 inhibitors, thus validating their potential hypoglycemic effects in a broader population. This approach not only increases the statistical robustness, but also improves the cost-effectiveness.

In mice, modulation of the microbiome by DPP-4 inhibitors such as vildagliptin, sitagliptin, and saxagliptin has been reported to contribute to their hypoglycemic effects, mainly through an increase in butyrate-producing bacteria [47,48,49]. In the case of *Drosophila*, a similar phenomenon may be plausible, as previous studies have indicated that their gut microbiome may influence their metabolic characteristics [50,51,52]. For example, *Drosophila* carrying *Acetobacteraceae*, unlike those carrying *Lactobacillus*, show a reduction in TAG and glycogen contents. It is likely that this phenomenon is due to the interaction between bacteria-mediated competition for dietary carbohydrates and stimulation of insulin signaling [50]. This will need to be corroborated in future research.

The choice of *Drosophila* as a model for DPP4 inhibitor screening is due to several compelling reasons. *Drosophila* is a well-established and extensively studied organism with a remarkable degree of genetic and metabolic conservation compared to humans [53]. This conservation extends to genes and processes relevant to diabetes and hyperglycemia-related conditions, making *Drosophila* an excellent model for this research [54]. Furthermore, the genetic manipulability of *Drosophila* is a key asset, as it allows precise and efficient modifications of DPP4 that enable researchers to identify genes and delve into the mechanistic underpinnings of diverse biological processes. These tools have been used extensively in the modeling of diseases related to insulin resistance, leading to the discovery of numerous candidate genes associated with diabetes and related diseases [55]. Meanwhile, due to the short lifespan and prolific reproductive rate of *Drosophila*, it is feasible to study the effects of DPP4 inhibitors over multiple generations [53]. *Drosophila* provides practicality, cost-effectiveness, and efficiency for large-scale experiments, enabling faster data acquisition compared to complex model systems [23].

Certainly, there are certain limitations to using *Drosophila* as a model for the study of DPP4 inhibitors. These limitations arise from the evolutionary divergence between *Drosophila* and humans, which may result in variations in drug responses due to differences in aspects of DPP4 function, such as substrate specificity and the extended cytoplasmic region of fruit fly DPP4. In addition, the simpler tissue structures of *Drosophila* compared to mammals pose challenges when investigating DPP4 within specific tissues or organs, as these structures do not precisely replicate human anatomy. Consequently, Drosophila may not comprehensively capture the intricacies of disease progression and its interactions with DPP4 inhibitors.

## 5. Conclusions

Our findings strongly advocate the use of *Drosophila* as a promising alternative model for the initial evaluation of hypoglycemic effects induced by novel drugs targeting DPP4 activity. This shift toward more efficient and ethical research methodologies is consistent with the broader goals of advancing pharmaceutical development while minimizing the use of experimental animals.

## Data Availability

The data that support the findings of this study are available from the corresponding author upon reasonable request.

## Supplementary Materials

The following supporting information can be downloaded at: https://www.mdpi.com/article/10.3390/biomedicines11113032/s1, Figure S1. Alignment of DPP4 orthologs across diverse species.
