# Supplementary material for: Drosophila as a Rapid Screening Model to Evaluate the Hypoglycemic Effects of Dipeptidyl Peptidase 4 (DPP4) Inhibitors: High Evolutionary Conservation of DPP4
Source: Biomedicines. 2023 Nov 12;11(11):3032. doi: 10.3390/biomedicines11113032 (PMC10669173; doi:10.3390/biomedicines11113032)
Supplement: Supplementary file 1 [file biomedicines-11-03032-s001.zip › biomedicines-2685215-supplementary.pdf]

— TM helix — DPPIV_N — Peptidase_S9 — DPPVI_rep

```
T._rubrum-Q5J6J3                  ............................................................
T._verrucosum-D4CZ59              ............................................................
T._equinum-A7UKV8                 ............................................................
T._tonsurans-B6V868               ............................................................
A._otae-A0S5V9                    ............................................................
A._oryzae-Q2UH35                  ............................................................
A._clavatus-A1CHP1                ............................................................
N._Fischeri-A1CX29                ............................................................
D._melanogaster-Q29R16_           ......................MSAAGWLDMNAALQNLDSPPETVKKARTKRRSSYAGRT
D._rerio-B5DDZ4                   ............................................................
P._textilis-A0A670YVH3            ............................................................
P._bivittatus-A0A9F2Q2Y6          ............................................................
C._porosus-A0A7M4FPI9             ............................................................
G._gallus-A0A1D5PJA5              ............................................................
L._coronata-A0A6J0IYQ4            ............................................................
M._domestica-K7DYU6               ............................................................
M._musculus-P28843                ............................................................
R._norvergicus-P14740             ............................................................
C._porcellus-A0A286XN52           ............................................................
H._glaber-A0A0P6J3T0              ............................................................
P._coquereli-A0A2K6GYP1           ............................................................
G._gorilla-G3SI68                 ............................................................
H._sapiens-P27487                 ............................................................
P._troglodytes-H2R124             ............................................................
N._leucogenys-G1QQ71              ............................................................
C._atys-A0A2K5LB00                ............................................................
M._mulatta-F6VRB0                 ............................................................
E._buettikoferi-A0A2Z5CWD4        ............................................................
C._perspicillata-A0A2Z5CWD9       ............................................................
R._aegyptiacus-A0A2Z5CWB8         ............................................................
A._planirostris-A0A2Z5CWB9        ............................................................
S._bilineata-A0A2Z5CWB7           ............................................................
R._ferrumequinum-A0A2Z5CWD5       ............................................................
M._gigas-A0A2Z5CWD8               ............................................................
L._africana-G3TVN4                ............................................................
E._caballus-A0A3Q2I3I7            ............................................................
A._melanoleuca-G1LG48             ............................................................
M._putorius-M3XN99                ............................................................
C._lupus-A0A8C0NCU9               CLTRAQAAGAAGLGGPGGGAGRASPGPPDLRPPPAPRAQFRARAEDEGECGRLPPRAVQV
F._catus-Q9N2I7                   ............................................................
C._hircus-A0A452FGS0              ............................................................
O._aries-W5P906                   ............................................................
B._taurus-P81425                  ............................................................
S._scrofa-P22411                  ............................................................
B._musculus-A0A8C0I050            ............................................................
D._leucas-A0A2Y9N2E9              ............................................................
P._macrocephalus-A0A2Y9EMH4       ............................................................
```

```
T._rubrum-Q5J6J3                  ............................................................
T._verrucosum-D4CZ59              ............................................................
T._equinum-A7UKV8                 ............................................................
T._tonsurans-B6V868               ............................................................
A._otae-A0S5V9                    ............................................................
A._oryzae-Q2UH35                  ............................................................
A._clavatus-A1CHP1                ............................................................
N._Fischeri-A1CX29                ............................................................
D._melanogaster-Q29R16_           TATAGVHGRGSIGTISSGIIKSFRERFLSNSSGDSGKYRKLRQRDAESGRMEASGSSSSG
D._rerio-B5DDZ4                   ............................................................
P._textilis-A0A670YVH3            ............................................................
P._bivittatus-A0A9F2Q2Y6          ............................................................
C._porosus-A0A7M4FPI9             ............................................................
G._gallus-A0A1D5PJA5              ............................................................
L._coronata-A0A6J0IYQ4            ............................................................
M._domestica-K7DYU6               ............................................................
M._musculus-P28843                ............................................................
R._norvergicus-P14740             ............................................................
C._porcellus-A0A286XN52           ............................................................
H._glaber-A0A0P6J3T0              ............................................................
P._coquereli-A0A2K6GYP1           ............................................................
G._gorilla-G3SI68                 ............................................................
H._sapiens-P27487                 ............................................................
P._troglodytes-H2R124             ............................................................
N._leucogenys-G1QQ71              ............................................................
C._atys-A0A2K5LB00                ............................................................
M._mulatta-F6VRB0                 ............................................................
E._buettikoferi-A0A2Z5CWD4        ............................................................
C._perspicillata-A0A2Z5CWD9       ............................................................
R._aegyptiacus-A0A2Z5CWB8         ............................................................
A._planirostris-A0A2Z5CWB9        ............................................................
S._bilineata-A0A2Z5CWB7           ............................................................
R._ferrumequinum-A0A2Z5CWD5       ............................................................
M._gigas-A0A2Z5CWD8               ............................................................
L._africana-G3TVN4                ............................................................
E._caballus-A0A3Q2I3I7            ............................................................
A._melanoleuca-G1LG48             ............................................................
M._putorius-M3XN99                ............................................................
C._lupus-A0A8C0NCU9               PPAPRPRTHPEVRQRGRGLPGKRGAALRQVAGRPARGPGRRTAARSPAPRSSLPGSAGAN
F._catus-Q9N2I7                   ............................................................
C._hircus-A0A452FGS0              ............................................................
O._aries-W5P906                   ............................................................
B._taurus-P81425                  ............................................................
S._scrofa-P22411                  ............................................................
B._musculus-A0A8C0I050            ............................................................
D._leucas-A0A2Y9N2E9              ............................................................
P._macrocephalus-A0A2Y9EMH4       ............................................................
```

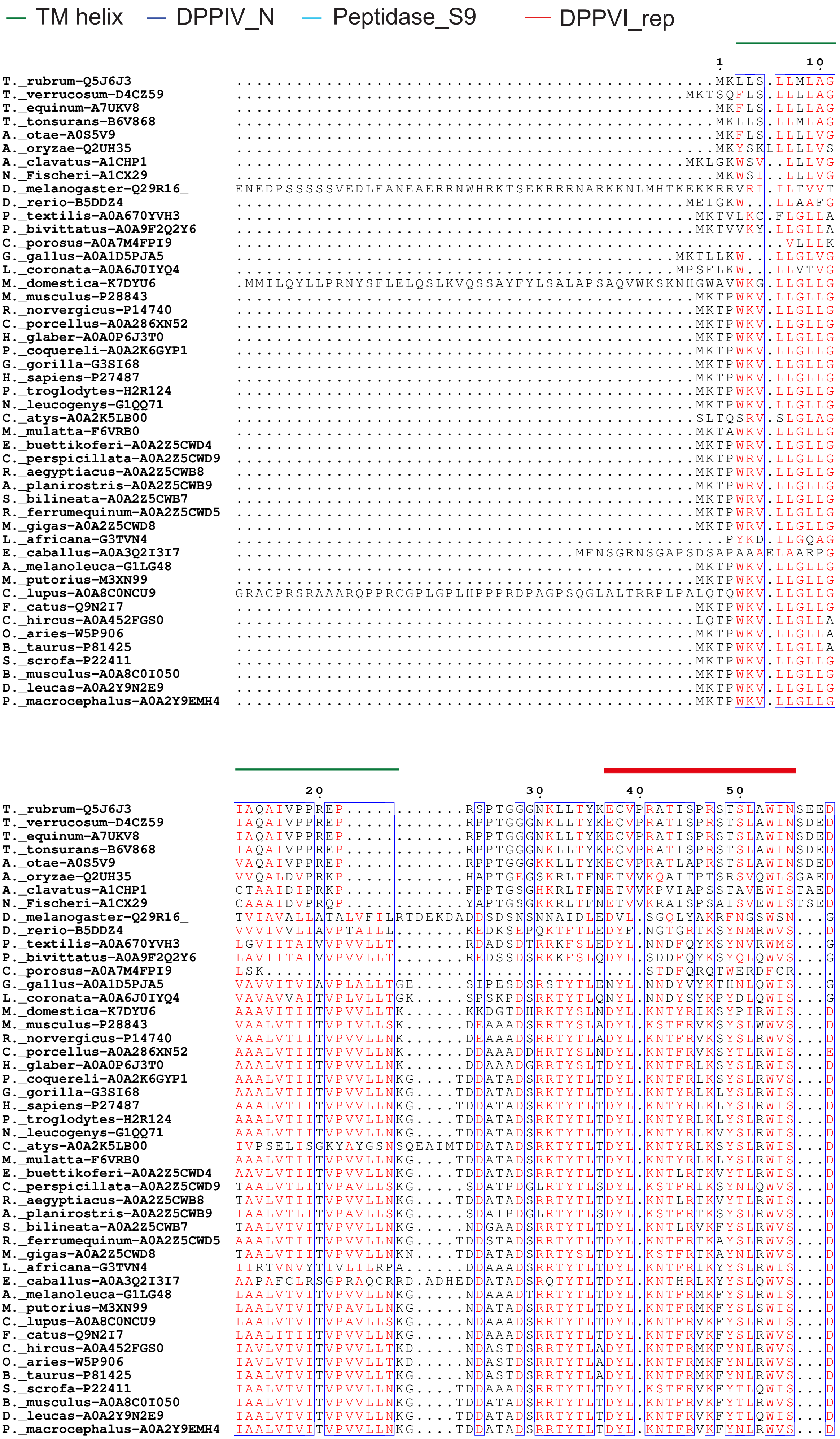
TM helix
DPPIV_N
Peptidase_S9
DPPVI_rep
T._rubrum-Q5J6J3
T._verrucosum-D4CZ59
T._equinum-A7UKV8
T._tonsurans-B6V868
A._otae-A0S5V9
A._oryzae-Q2UH35
A._clavatus-A1CHP1
N._Fischeri-A1CX29
D._melanogaster-Q29R16_
D._rerio-B5DDZ4
P._textilis-A0A670YVH3
P._bivittatus-A0A9F2Q2Y6
C._porosus-A0A7M4FPI9
G._gallus-A0A1D5PJA5
L._coronata-A0A6J0IYQ4
M._domestica-K7DYU6
M._musculus-P28843
R._norvergicus-P14740
C._porcellus-A0A286XN52
H._glaber-A0A0P6J3T0
P._coquereli-A0A2K6GYP1
G._gorilla-G3SI68
H._sapiens-P27487
P._troglodytes-H2R124
N._leucogenys-G1QQ71
C._atys-A0A2K5LB00
M._mulatta-F6VRB0
E._buettikoferi-A0A2Z5CWD4
C._perspicillata-A0A2Z5CWD9
R._aegyptiacus-A0A2Z5CWB8
A._planirostris-A0A2Z5CWB9
S._bilineata-A0A2Z5CWB7
R._ferrumequinum-A0A2Z5CWD5
M._gigas-A0A2Z5CWD8
L._africana-G3TVN4
E._caballus-A0A3Q2I3I7
A._melanoleuca-G1LG48
M._putorius-M3XN99
C._lupus-A0A8C0NCU9
F._catus-Q9N2I7
C._hircus-A0A452FGS0
O._aries-W5P906
B._taurus-P81425
S._scrofa-P22411
B._musculus-A0A8C0I050
D._leucas-A0A2Y9N2E9
P._macrocephalus-A0A2Y9EMH4

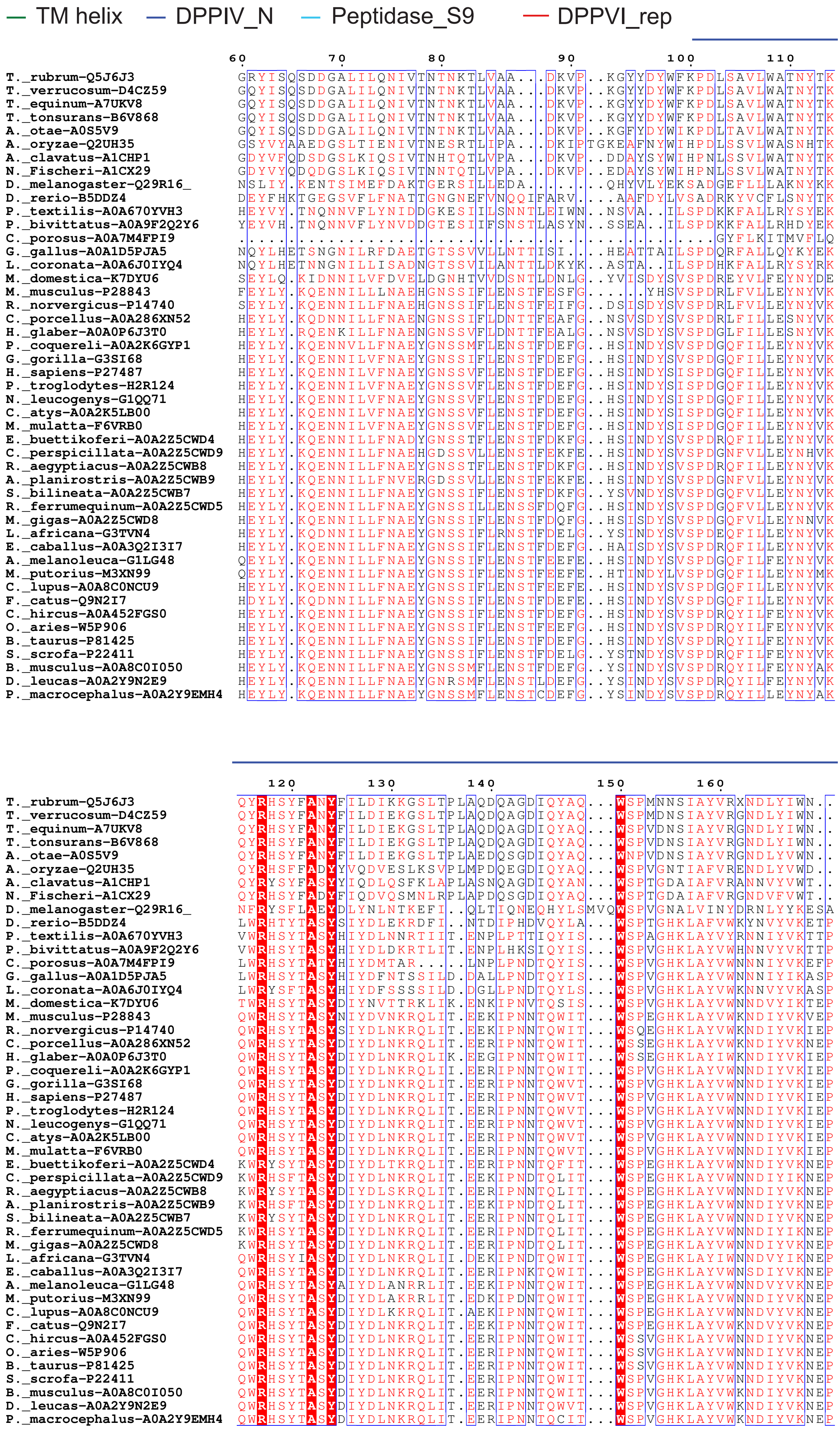
TM helix
DPPIV_N
Peptidase_S9
DPPVI_rep

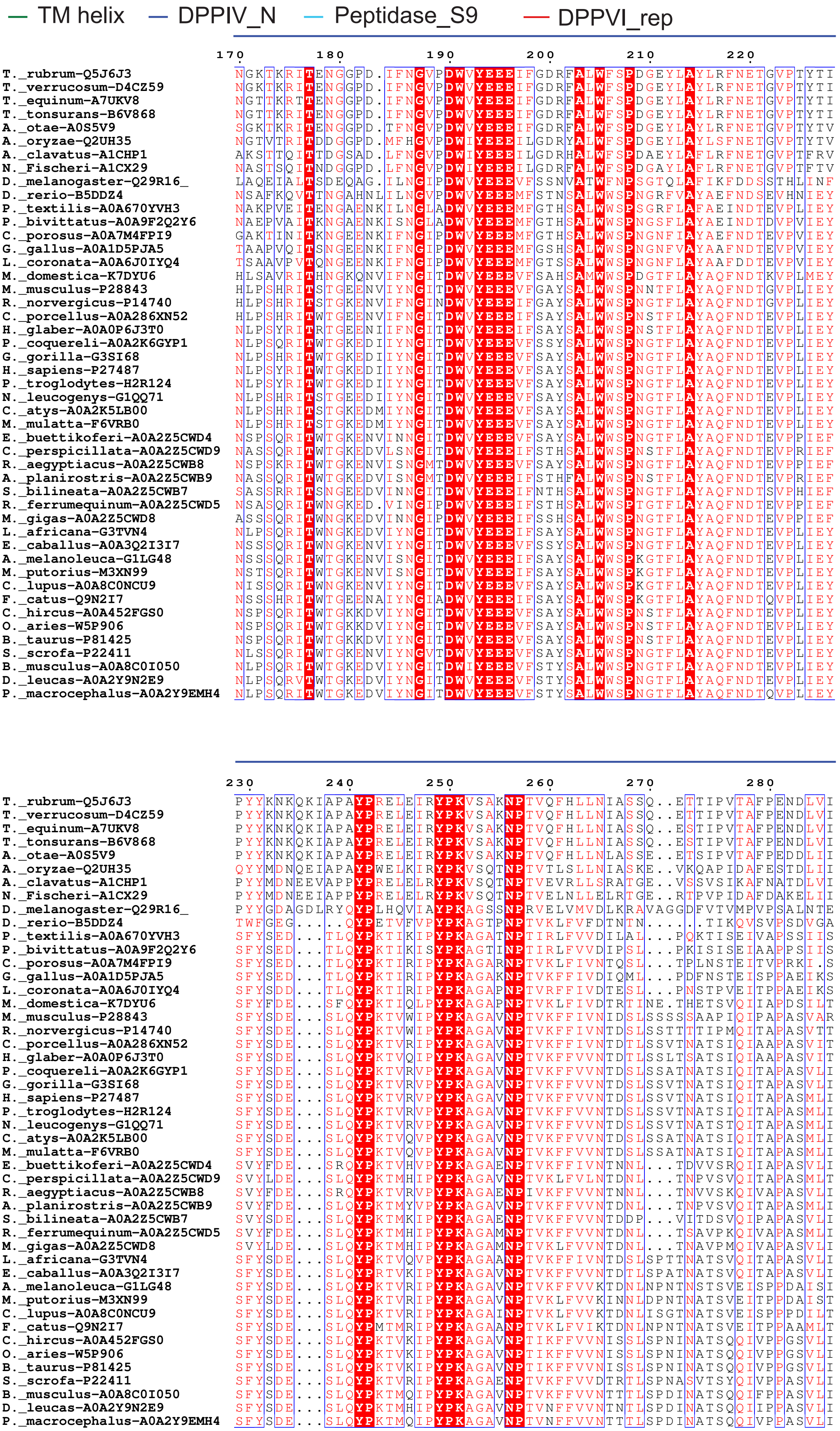

— TM helix — DPPIV_N — Peptidase_S9 — DPPVI_rep
170 180 190 200 210 220
230 240 250 260 270 280
T._rubrum-Q5J6J3
T._verrucosum-D4CZ59
T._equinum-A7UKV8
T._tonsurans-B6V868
A._otae-A0S5V9
A._oryzae-Q2UH35
A._clavatus-A1CHP1
N._Fischeri-A1CX29
D._melanogaster-Q29R16_
D._rerio-B5DDZ4
P._textilis-A0A670YVH3
P._bivittatus-A0A9F2Q2Y6
C._porosus-A0A7M4FPI9
G._gallus-A0A1D5PJA5
L._coronata-A0A6J0IYQ4
M._domestica-K7DYU6
M._musculus-P28843
R._norvergicus-P14740
C._porcellus-A0A286XN52
H._glaber-A0A0P6J3T0
P._coquereli-A0A2K6GYP1
G._gorilla-G3SI68
H._sapiens-P27487
P._troglodytes-H2R124
N._leucogenys-G1QQ71
C._atys-A0A2K5LB00
M._mulatta-F6VRB0
E._buettikoferi-A0A2Z5CWD4
C._perspicillata-A0A2Z5CWD9
R._aegyptiacus-A0A2Z5CWB8
A._planirostris-A0A2Z5CWB9
S._bilineata-A0A2Z5CWB7
R._ferrumequinum-A0A2Z5CWD5
M._gigas-A0A2Z5CWD8
L._africana-G3TVN4
E._caballus-A0A3Q2I3I7
A._melanoleuca-G1LG48
M._putorius-M3XN99
C._lupus-A0A8C0NCU9
F._catus-Q9N2I7
C._hircus-A0A452FGS0
O._aries-W5P906
B._taurus-P81425
S._scrofa-P22411
B._musculus-A0A8C0I050
D._leucas-A0A2Y9N2E9
P._macrocephalus-A0A2Y9EMH4

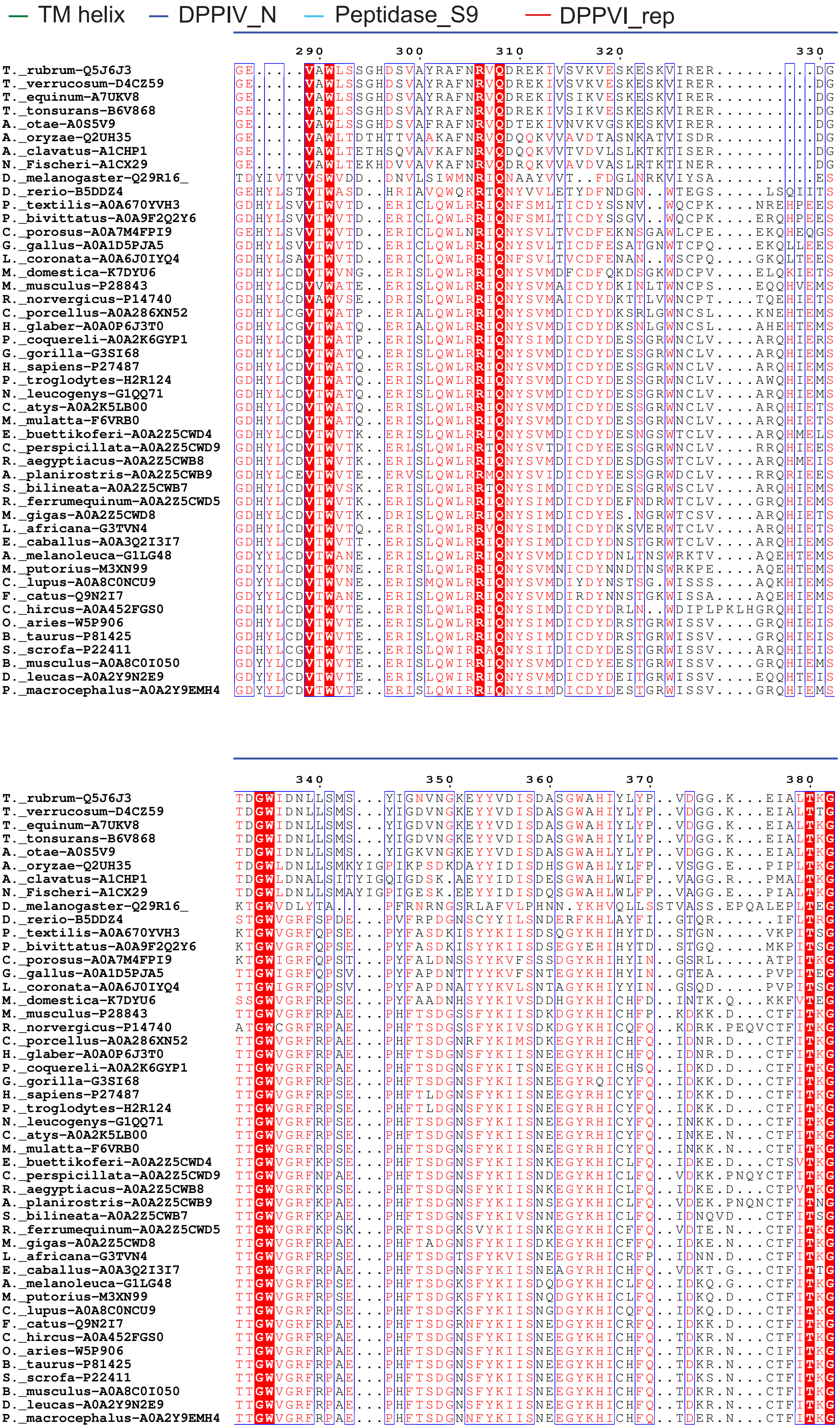
TM helix
DPPIV_N
Peptidase_S9
DPPVI_rep

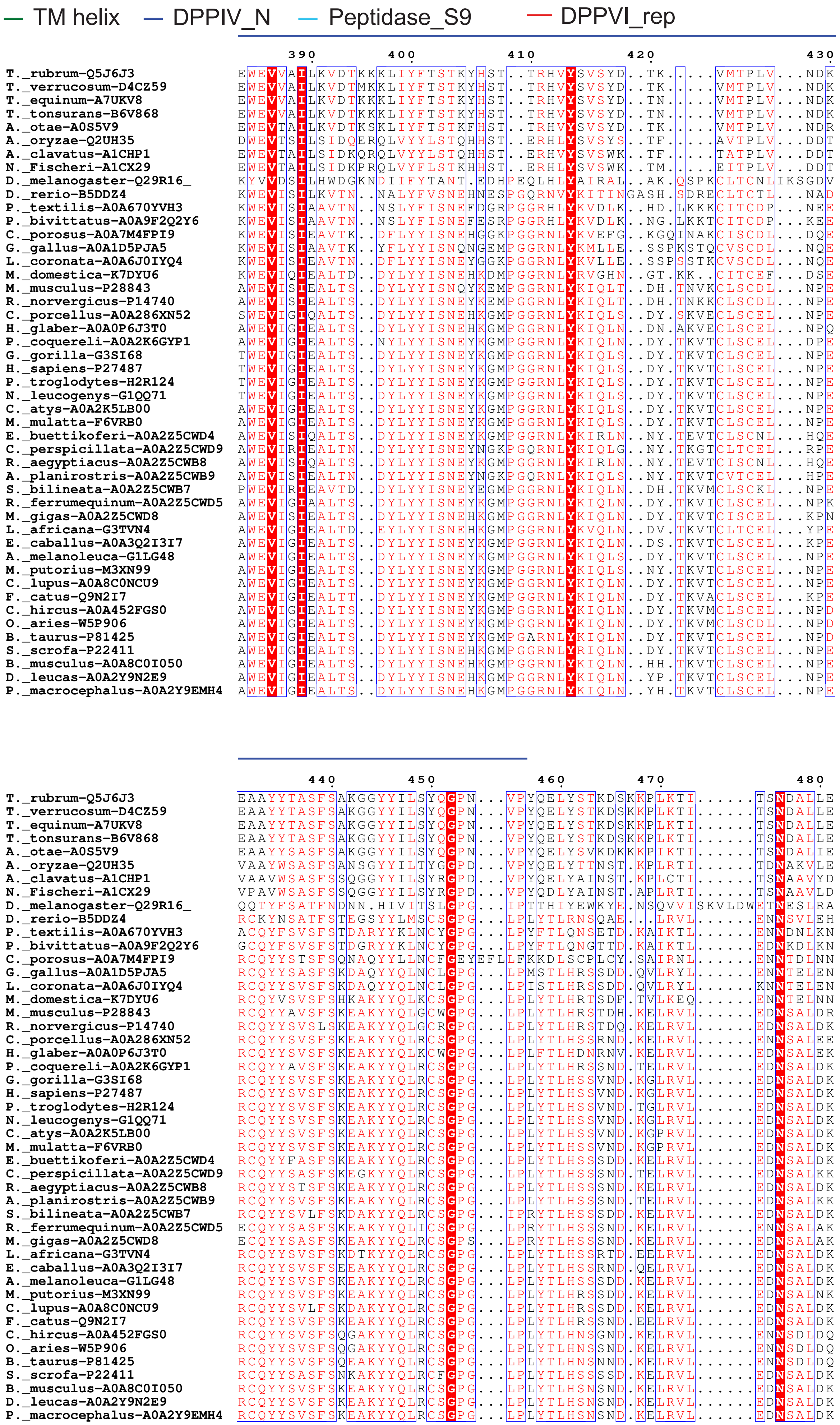
TM helix
DPPIV_N
Peptidase_S9
DPPVI_rep

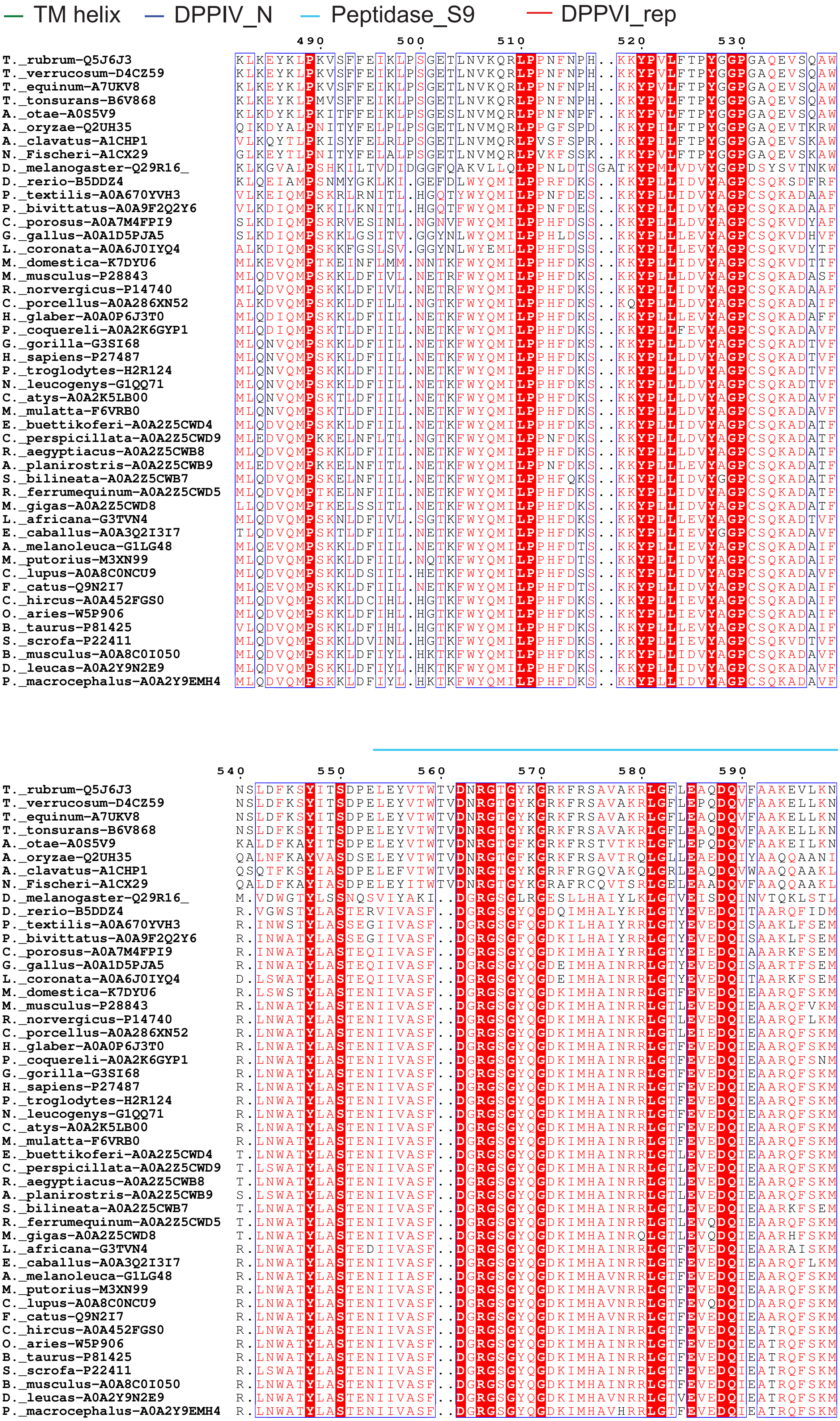
TM helix
DPPIV_N
Peptidase_S9
DPPVI_rep

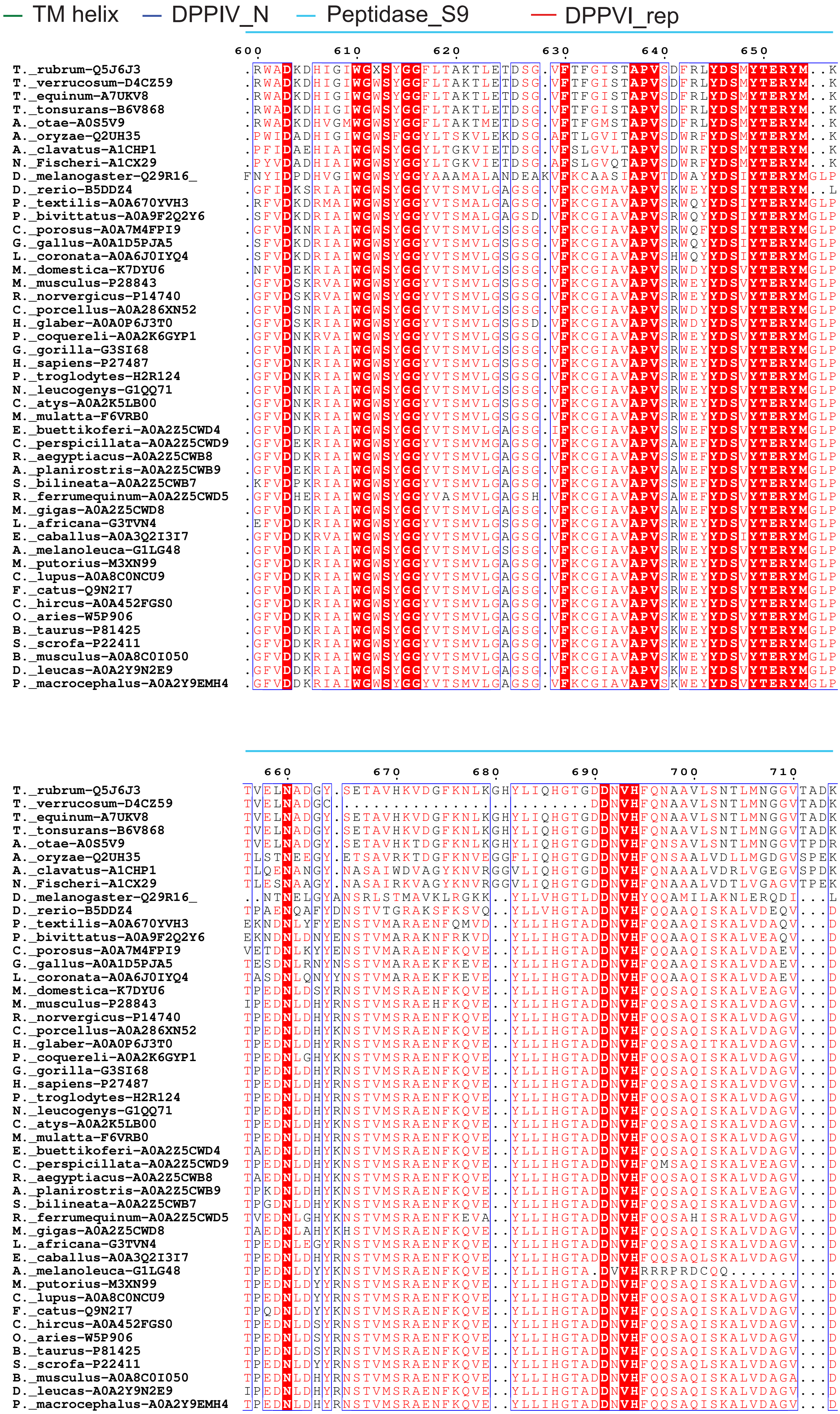
TM helix
DPPIV_N
Peptidase_S9
DPPVI_rep
600
610
620
630
640
650
660
670
680
690
700
710
T._rubrum-Q5J6J3
T._verrucosum-D4CZ59
T._equinum-A7UKV8
T._tonsurans-B6V868
A._otae-A0S5V9
A._oryzae-Q2UH35
A._clavatus-A1CHP1
N._Fischeri-A1CX29
D._melanogaster-Q29R16_
D._rerio-B5DDZ4
P._textilis-A0A670YVH3
P._bivittatus-A0A9F2Q2Y6
C._porosus-A0A7M4FPI9
G._gallus-A0A1D5PJA5
L._coronata-A0A6J0IYQ4
M._domestica-K7DYU6
M._musculus-P28843
R._norvergicus-P14740
C._porcellus-A0A286XN52
H._glaber-A0A0P6J3T0
P._coquereli-A0A2K6GYP1
G._gorilla-G3SI68
H._sapiens-P27487
P._troglodytes-H2R124
N._leucogenys-G1QQ71
C._atys-A0A2K5LB00
M._mulatta-F6VRB0
E._buettikoferi-A0A2Z5CWD4
C._perspicillata-A0A2Z5CWD9
R._aegyptiacus-A0A2Z5CWB8
A._planirostris-A0A2Z5CWB9
S._bilineata-A0A2Z5CWB7
R._ferrumequinum-A0A2Z5CWD5
M._gigas-A0A2Z5CWD8
L._africana-G3TVN4
E._caballus-A0A3Q2I3I7
A._melanoleuca-G1LG48
M._putorius-M3XN99
C._lupus-A0A8C0NCU9
F._catus-Q9N2I7
C._hircus-A0A452FGS0
O._aries-W5P906
B._taurus-P81425
S._scrofa-P22411
B._musculus-A0A8C0I050
D._leucas-A0A2Y9N2E9
P._macrocephalus-A0A2Y9EMH4

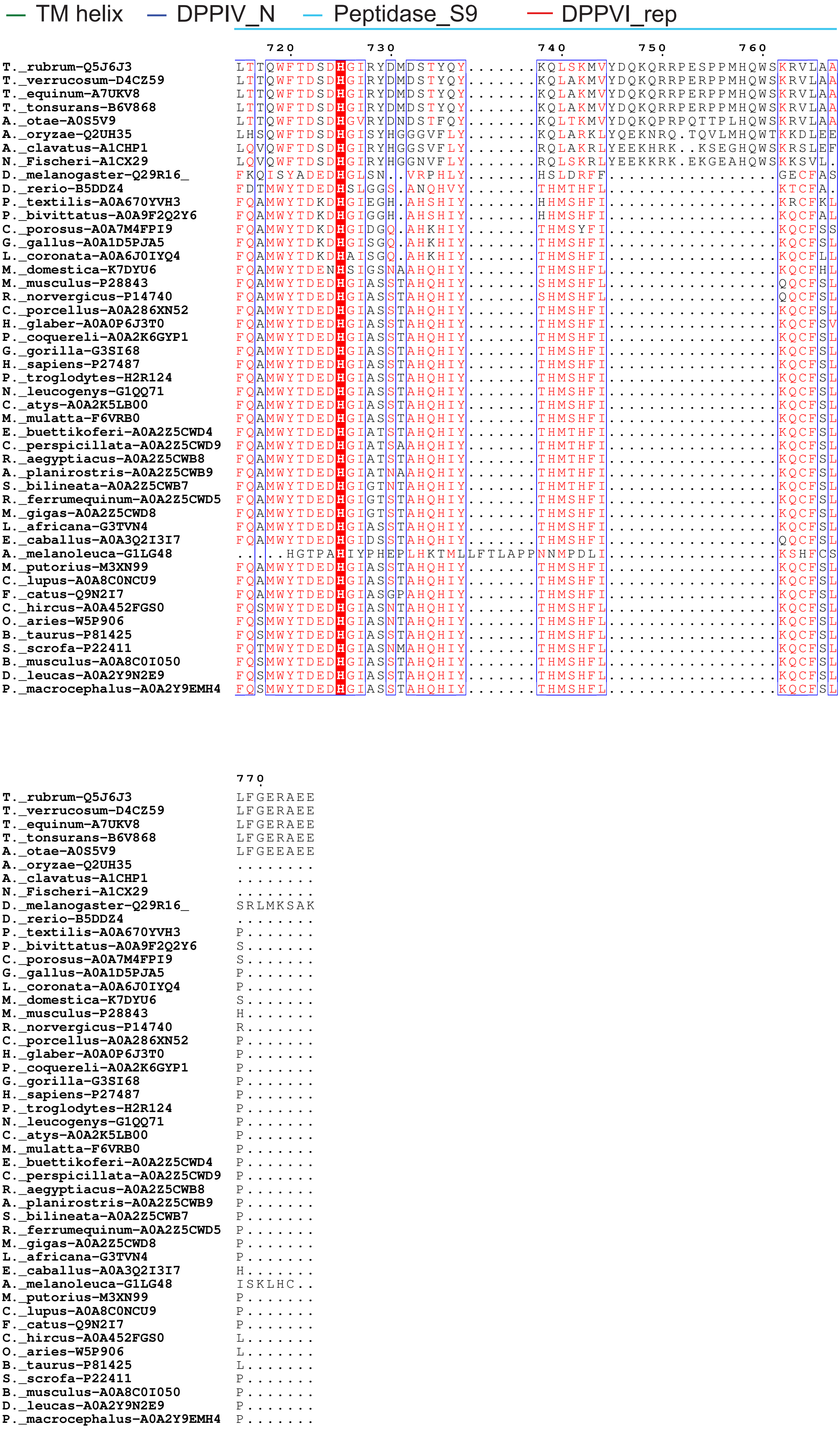

Figure S1. Alignment of DPP4 orthologs across diverse species.
